# Supplementary material for: Genome-Wide Identification and Comprehensive Analysis of the GASA Gene Family in Peanuts (Arachis hypogaea L.) under Abiotic Stress
Source: Int J Mol Sci. 2023 Dec 4;24(23):17117. doi: 10.3390/ijms242317117 (PMC10707693; doi:10.3390/ijms242317117)
Supplement: Supplementary file 1 [file ijms-24-17117-s001.zip › ijms-2690695-supplementary.pdf]

Table S1. List of primers used for gene expression analysis in this study

S.No	Accession/Gene	Forward Primer	Reverse Primer	GC	Tm	Product Size
Primer-1	XG1GJV/AhGASA39	CTTTGTTGGAACCAGCCACT	AGGTGCCAGAAGGAACACAC	50/55	60	150
Primer-2	VJ6NUA/AhGASA31	TTATGGCAAAAGAGGCTGCT	ACGCTTGGTCTTCCAGTTGT	45/50	59.87	233
Primer-3	FK6HB9/AhGASA1	GCTTCTCGGAGTGTTCTTGC	TCGGTCTTGAGTGCAAACTG	55/50	60	194
Primer-4	RUJ0QA/AhGASA22	CCACCCACACCTTATCATCC	TTCGCGGTTACCGTAAGTTC	50	60	216
Primer-5	VES4LD/AhGASA25	AAAGTGTGCAAGGAGGTGCT	TTAAGGGCACTTTGGTTTGC	50	60	172
Primer-6	AVE3D2/AhGASA4	TGATGCTGCTACCACAGAGG	ACCAGGAGGCACACATAAGC	55	60	217
Primer-7	6SHE48/AhGASA9	TTATGGCAAAAGAGGCTGCT	CGCTTGGTCTTCCAGTTGTT	48	59	232
Primer-8	FJ610L/AhGASA21	ATGGACCTGGGAGTGTCAAG	TGTTGTAGCAAGGGCAAACA	55/45	59	203
Primer-9	F1TUFL/AhGASA40	GACCTGGCAGTCTCAAGAGC	GCATTTTGGTCCTCCTTCCT	55	60	200
Primer-10	PW1K78/AhGASA19	ATTGCAGACACCAATGCAAA	AAGTTCCTGGCGGTACACAC	45	60	226
Primer-11	YB2114/AhGASA2	TTTGTGGATTGTGGAGCAAA	TGGGTTTGTTGCCATGAGTA	45	60	181
Primer-12	TJLQ2T/AhGASA13	GGATGTCTCAATGGCTGGTT	AGGGCACTCGTCCTTATTCC	50	60	160
Primer-13	JGC745.1/AhGASA8	ATTCTCCAATGTGGCTGAGG	GGAGGAACACACAGGCATTT	50	60	153
Primer-14	SY561U.1/AhGASA30	GGATGTCTCAATGGCTGGTT	AGGGCACTCGTCCTTATTCC	52	60	160
Primer-15	C0SGC9.1/AhGASA27	TGTCTTTGCTCCTCCTCCAT	ACGCTCTCTTGCACATCCTT	50	60	159
Primer-16	XBXR9Z.1/AhGASA29	AGATGCAGCAAAACATCACG	TTAAGGGCACTTGCGTCTG	45/52	60	159
Primer-17	CH1EP2.1/AhGASA20	TTTGTGGATTGTGGAGCAAA	GGGCTTGTTGCCATGAGTA	40/52	60	180
Primer-18	RF89RI.1/AhGASA17	GATGCAGTAAGGCAGGAAGG	GGCACTTCAATTTTCCTCCA	55/45	60	153

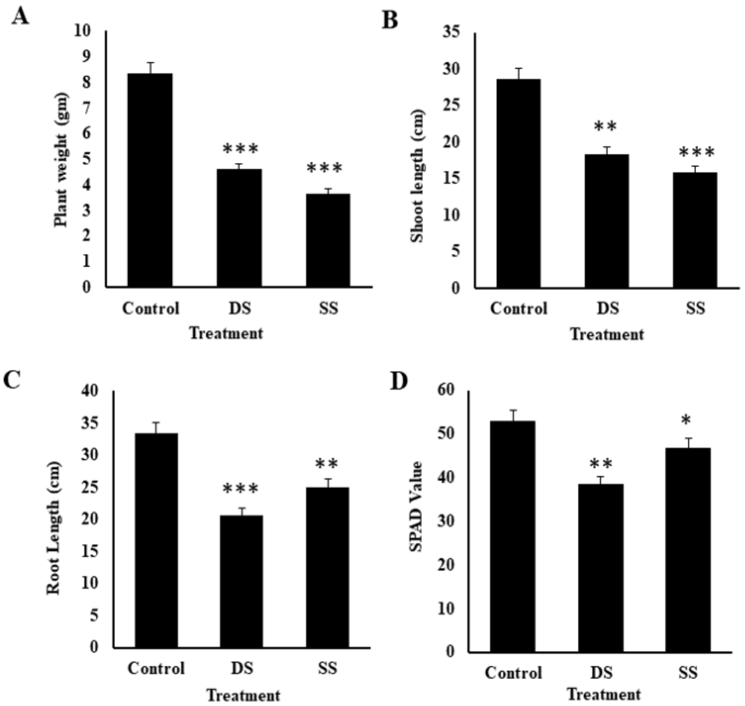

Figure S1. Plant attributes under drought, salinity, and control conditions. (A) Plant weight, (B) plant shoot length, (C) root length, and (D) chlorophyll content (SPAD value). Abbreviations: control, well water; DS, drought stress; and SS, salt stress. Data are presented as the mean ± standard error of three biological replicates. Means were analyzed for significant differences using Student's *t*-test. Asterisks (*) indicate significant differences at a 5% level (***$p < 0.001$, **$p < 0.01$, *$p < 0.05$).
